# Perceptions of Home Telemonitoring Use Among Patients With Chronic Obstructive Pulmonary Disease: Qualitative Study

**DOI:** 10.2196/16343

**Published:** 2020-06-03

**Authors:** Sara Lundell, Mari Modig, Åsa Holmner, Karin Wadell

**Affiliations:** 1 Division of Physiotherapy Department of Community Medicine and Rehabilitation Umeå University Umeå Sweden; 2 Department of Epidemiology and Global Health Umeå University Umeå Sweden; 3 Department of Radiation Sciences Umeå University Umeå Sweden; 4 Division of Medicine Department of Public Health and Clinical Medicine Umeå University Umeå Sweden

**Keywords:** COPD, qualitative content analysis, eHealth, chronic disease, home-based care, empowerment, information and communication technology

## Abstract

**Background:**

Chronic obstructive pulmonary disease (COPD) is a major health problem and an economic burden globally. There is growing interest in how electronic health (eHealth) can be used to provide efficient health care. Telemonitoring, where the patient’s health-related data is transmitted to a health care provider, can be used to detect early signs of exacerbations. A successful implementation of telemonitoring systems into clinical practice requires in-depth knowledge of the users’ preferences.

**Objective:**

The aim of this study was to explore perceptions of the use of a home telemonitoring system among patients with COPD.

**Methods:**

Semistructured individual interviews were carried out with 8 women and 5 men who were participants in a project aimed at developing and evaluating a telemonitoring system. The web-based telemonitoring system measured pulmonary function, subjective symptoms, and oxygen saturation. Participants were interviewed after having used the system for 2-4 months. Interview transcripts were analyzed with qualitative content analysis.

**Results:**

The analysis resulted in the theme *A transition toward increased control and security* and four categories: using with (in)security, affecting technical concern or confidence, providing easy access to health care, and increasing control over the disease. The participants reported various perceptions of using the telemonitoring system. They expressed initial feelings of insecurity, both in terms of operating the system and in terms of their disease. However, the practical management of the telemonitoring system became easier with time; the participants gradually gained confidence and improved their self-management. New technology was perceived as an important complement to existing health care, but the importance of maintaining a human contact in real life or through the telemonitoring system was emphasized.

**Conclusions:**

This study captured a transition among the participants from being insecure and experiencing technical concerns to acquiring technical confidence and improving disease management. Telemonitoring can be a valuable complement to health care, leading to increased self-knowledge, a sense of security, and improved self-management. Suggestions to improve the further development and implementation of telemonitoring systems include better patient education and the involvement of end users in the technical development process. Additional research is needed, particularly in the design of user-friendly systems, as well as in developing tools to predict which patients are most likely to find the equipment useful, as this may result in increased empowerment, improved quality of life, reduced costs, and a contribution to equity in health.

## Introduction

Chronic obstructive pulmonary disease (COPD) is a major public health problem [1]. According to the World Health Organization, COPD is the third-leading cause of death worldwide [2]. Although chronic progressive dyspnea is the most characteristic symptom, COPD is considered a multicomponent disease with many systemic consequences, for example, cardiovascular diseases and decreased physical capacity [1]. In addition, over 80% of patients with COPD have at least one chronic comorbidity [3].

Patients with COPD often present with acute exacerbations (AEs) [4]. An AE is defined as an acute worsening of respiratory symptoms resulting in additional therapy [5]. Severe AEs requiring hospitalization result in a decrease in physical activity level and physical capacity with decreased muscle strength, increased impairment, and increased mortality [4,6]. Health care costs for COPD, in which costs for AEs are a strong contributing factor, are 68% higher than for people without COPD [7]. It is, therefore, of great importance to find strategies to reduce the number and severity of AEs. The annual societal costs of COPD in Sweden were estimated to be €1.5 billion in 2010 [8]. Since a history of previous AEs is an important predictor of future AEs [9], the health care target should be focused on the prevention of the first AE [1].

There is growing interest in how electronic health (eHealth) can be used to provide efficient health care. One example is telemonitoring, defined as the use of information and communication technology for the automated transmission of health-related data from a patient’s home to a health care provider. In this way, clinicians can be alerted if any abnormal parameters occur and can take immediate action to prevent complications and in-hospital care [10]. Previous studies on telemonitoring for patients with COPD have shown that telemonitoring can detect exacerbations and reduce the number of hospitalizations, as well as improve their mental-health quality of life, while the results on other health care utilization outcomes are inconsistent [11,12]. Telemonitoring seems to reduce health care costs [12,13]; however, no effect on mortality has been found [12]. A systematic review of mainly quantitative studies has shown that patients with COPD were satisfied and experienced that the telemonitoring systems were a help in monitoring their disease [14]. Reported perceptions were mixed in previous qualitative studies, ranging from experiences of the telemonitoring systems being reassuring, encouraging, and improving the self-management of the health condition to perceptions that the systems were disturbing and caused worry [14-19]. However, some of these studies have combined telemonitoring with patient education, exercise, or an action plan including medication. Consequently, few qualitative studies have explored how people with COPD experience the sole use of telemonitoring in addition to conventional care.

Some studies using telemonitoring in patients with COPD have reported a low adherence and a high dropout rate, often because of the telemonitoring system itself [14]. It is essential to understand the patients’ perspectives in order to recognize barriers to and enablers of accepting new technology for telemonitoring systems to be successfully implemented into clinical practice. Hence, there is a need for further research to ensure that telemonitoring systems fit the needs and preferences of the users. The aim of this study was, therefore, to explore perceptions of the use of a home telemonitoring system in patients with COPD. 

## Methods

### Study Design

This study had a qualitative research design with semistructured interviews. In order to improve transparency and strengthen transferability [20], the study was conducted and reported according to the Standards for Reporting Qualitative Research, a 21-item checklist by O'Brien et al [21].

### Setting

**Figure 1:**
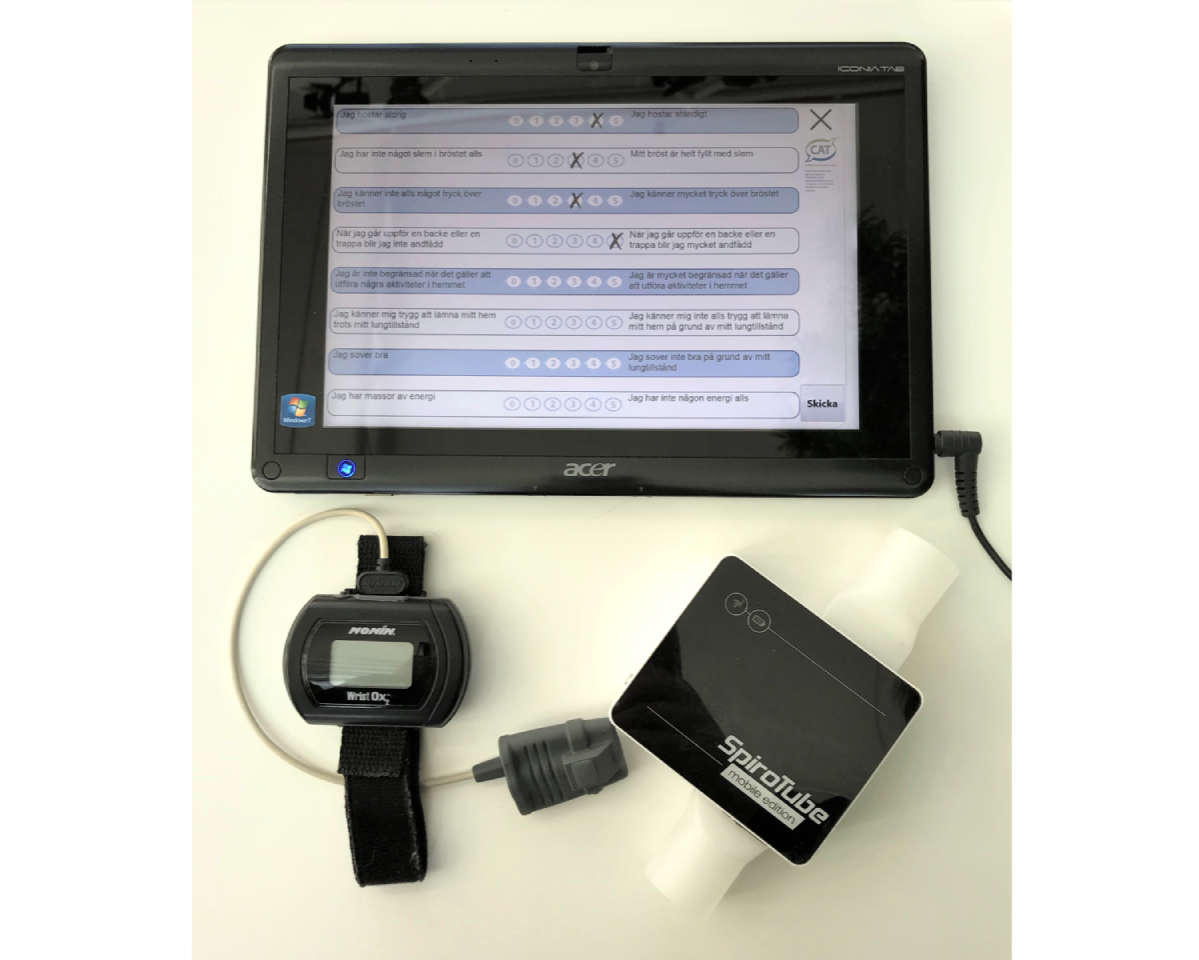
The telemonitoring system: a tablet computer with the telemonitoring system (top), a pulse oximeter (bottom left), and a spirometer (bottom right).

This qualitative study is part of a telemonitoring project aimed at introducing and evaluating a web-based telemonitoring system that measures pulmonary function (ie, inspiratory capacity [IC] and forced expiratory volume in 1 second [FEV_1_]), subjective symptoms using the COPD assessment test (CAT), [1] and oxygen saturation (see Figure 1) [22,23]. The telemonitoring project was conducted in Västerbotten county in northern Sweden, a large and sparsely populated area with long distances to health care facilities for many inhabitants. The aim of the telemonitoring project was to evaluate the ability of the system to detect early signs of AEs in patients with COPD. The plan was to connect the system to health care professionals in a later stage, to detect and act on changes in symptoms indicating exacerbations. The participants used the system to perform measurements at home twice a day, 3 days a week, for 4-6 months. The system provided direct visual feedback showing heart rate, oxygen saturation, and a visual cue indicating satisfactory airflow during IC measurements. The complete spirometry curve was shown during the FEV_1_ procedure, but no values were calculated. No historic data were shown to the participants. Recorded data were stored locally and were automatically transmitted to the study center using the mobile 4G network. Technical support was provided to the participants during the study by one of the system developers, an electronics engineer. To evaluate if exacerbations could actually be detected, the researchers did not act on the data (ie, did not intervene with the participants during the study) unless an obvious mistake was detected. This decision was made to obtain unaffected data during the study to evaluate if exacerbations can actually be detected. The participants were well informed about this strategy and were instructed to contact their usual health care provider if they needed medical support.

### Sampling and Participants

Participants for the interview study were recruited from a group of patients participating in the telemonitoring project [23] who were being treated for COPD at the Department of Medicine, Division of Respiratory Medicine and Allergy, University Hospital, Umeå, Sweden. The participants were approached separately for the interview study and informed that their decision would not influence their participation in the telemonitoring project or their usual care. A convenience sampling method was used, striving for a broad representation regarding age, gender, disease severity, and computer experience. Inclusion criteria for the interview study were as follows: COPD 2-4 according to the Global Initiative for Chronic Obstructive Lung Disease (GOLD) classification [1], aged 40 years or older, resident of Västerbotten county, included in the telemonitoring project, able to understand and speak Swedish, and have used the system for approximately 3 months. The exclusion criterion was comorbidity that could prevent participation in the interview (eg, severe psychiatric disorder). Eventually, all participants in the telemonitoring project [23] were invited and accepted to participate in the interview study.

Before enrollment in the telemonitoring project, the participants performed standard tests and received oral and written information as well as practical training in using the telemonitoring system [23]. Background data for this study was collected using the modified Medical Research Council (mMRC) dyspnea scale [24], the Hospital Anxiety and Depression Scale (HADS) [25], and a spirometer test. A question about computer experience was also included; it was answered on a scale from 1 (no experience) to 10 (very experienced). In addition, a question about how sure the participants were about their ability to use the system was answered on a scale from 1 (not sure at all) to 10 (very sure). A total of 13 participants were included: 5 men (38%) and 8 women (62%) (see Table 1).

**Table 1:** Characteristics of participants included in the interview study.

Characteristic		Value (N=13)	
**Gender, n (%)**			
	Women		8 (62)
	Men		5 (38)
Age (years), median (min-max)		70 (48-80)	
**GOLD^a^ classification grade, n (%)**			
	2		8 (62)
	3		3 (23)
	4		2 (15)
FEV_1_^b^/FVC^c^ (%), median (min-max)		54 (25-65)	
FEV_1_ (% predicted), median (min-max)		50 (21-68)	
**mMRC^d,e^ dyspnea scale (score), n (%)**			
	1		3 (23)
	2		5 (38)
	3		1 (8)
	4		4 (31)
**HADS^e,f^(score), median (min-max)**			
	Anxiety		5 (0-10)
	Depression		4 (1-12)
Computer experience^g^, median (min-max)		6 (1-10)	
Expected ability to use the system^h^, median (min-max)		8 (5-10)	

^a^GOLD: Global Initiative for Chronic Obstructive Lung Disease.

^b^FEV_1_: forced expiratory volume in 1 second.

^c^FVC: forced vital capacity.

^d^mMRC: modified Medical Research Council.

^e^A higher number indicates increased breathlessness, anxiety, or depression.

^f^HADS: Hospital Anxiety and Depression Scale.

^g^Computer experience was rated on a scale from 1 (no experience) to 10 (very experienced).

^h^Expected ability was rated on a scale from 1 (not sure at all) to 10 (very sure).

### Data Collection

The semistructured interviews were performed between March 2014 and January 2016, after 2-4 months’ use of the telemonitoring system. The interviews were conducted by SL, typically in the participants’ homes (7/13, 54%); the rest were conducted by telephone (4/13, 31%) or at the hospital (2/13, 15%). A total of 2 out of 13 (15%) participants chose to have a family member present during the interview. The interview guide included questions about the participants’ experiences of using the system, areas of improvement, and their contact with health care providers (see Textbox 1). Each question area started with an open-ended question, then follow-up questions, and prompts were used when needed. The interviews lasted between 10 and 46 minutes, most being around 30 minutes; were audio-recorded; and were transcribed verbatim by SL or a professional transcriber.

**Interview guide based on question areas.**

Experiences of using the system:

- What are your experiences of using the system?
- What has been good and bad?
- Has the perception of your symptoms changed?

Areas of improvement:

- What could be made better?
- How should an ideal system work?

Contact with health care:

- What health care contacts do you have?
- Have your contacts with health care providers changed?

### Data Analysis

Qualitative content analysis with an inductive approach was used to analyze the data, according to the procedure described by Graneheim and Lundman [20]. The analysis was performed by MM in close collaboration with SL. The transcribed interviews were read several times to get a sense of the material, and the transcriptions were then inductively coded. The codes were compared to find similarities and differences and then grouped into categories and subcategories. This process was iterative, going back and forth, comparing categories, codes, and interview texts. Finally, an interpretative theme that could be identified across categories was abstracted from the underlying (ie, latent) content. The analysis was discussed and re-evaluated several times between MM, SL, and KW In order to achieve agreement among the researchers [20]. The three authors involved in the analysis were all physiotherapists but still contributed with different perspectives. The software Open Code 4.03 (Umeå University, Sweden) [26] was used as a tool in the analysis process to facilitate administrating the interviews, codes, and quotes.

### Ethical Considerations

The study was approved by the Regional Ethical Review Board in Umeå (Dnr: 2013-187-31Ö) and followed the principles of the Declaration of Helsinki. Written informed consent was obtained from all participants. Participant confidentiality was ensured throughout the whole process, from data collection to the presentation of the findings.

## Results

### A Transition Toward Increased Control and Security

The theme *A transition toward increased control and security* was formulated during the analysis. Participants expressed initial feelings of insecurity, both in practical aspects of using the telemonitoring system as well as regarding their disease. They experienced increased self-knowledge and deeper understanding of symptom variability when the system confirmed their health status, but it was also important to receive support from health care professionals. The practical management of the system became easier with time and participants reported an increase in technical confidence. The system was considered an important complement to existing health care, despite its lack of feedback on measurements taken during the study. However, maintaining human contact was considered essential. Overall, the use of the telemonitoring system improved the participants’ perceived self-management over time.

The theme comprises four categories and 10 subcategories (see Table 2). The categories are presented in the following section with the subcategories embedded in the text, exemplified with quotes from the participants.

**Table 2:** Categories and subcategories of the theme A transition toward increased control and security.

Categories	Subcategories
Using with (in)security	Insecurity in management Need for information Easy to use
Affecting technical concern or confidence	Negative feelings about technology Increased technical confidence
Providing easy access to health care	Complement to health care Importance of human contact
Increasing control over the disease	Feeling reassured Increased awareness Management of the disease

### Using With (In)Security

This category represents the participants’ experiences of using the telemonitoring system, reflecting both insecurity and satisfaction. Participants expressed both insecurity and the need for more support about the management of both the technology and tests, while at the same time describing the system as easy to use.

Participants experienced *insecurity in management* of the system and a fear of damaging it. Questions arose regarding whether the information was sent or not, expressed as follows:

But the only thing I’ve maybe felt sometimes...I wonder whether they received the information?

Woman, 58 years

In addition, participants found it worrying not knowing if the system would work properly or not:

So that’s always at the back of your mind—I hope it works today.

Woman, 71 years

Participants also reported that when technical failures did occur, feelings of frustration, anger, irritation, and disappointment arose. Experiencing technical failures led to insecurity and questions about whether there was an error in the system itself, or if the failure was caused by inadequate management. Furthermore, a need for simplification of the system was requested. First, the language was sometimes perceived as difficult to understand, which made the task of interpreting and answering questions challenging. Second, it was considered time-consuming and physically demanding to perform the tests, especially in the mornings; this was expressed as follows:

It is quite exerting when you try so hard, I can get chest pains afterwards.

Woman, 48 years

This impacted daily life in a negative way. Participants reported a feeling of being “chained” to the system, since thoughts about the system and remembering to perform the tests were always present. This could lead to a feeling of being “fed up” and a wish for “a break” from the system. Since the telemonitoring system was considered “bulky,” it was perceived as difficult to take when traveling.

Participants expressed a *need for information*, as insufficient information about the system was raised as another area for improvement. A common problem identified was that information once provided was easy to forget. Hence, the desire for repeated information, both orally and in writing, and the desire to test the system under supervision for a longer period of time were also expressed. The need for more information justifying the use of the telemonitoring system was needed, since the purpose of using it was unclear in some cases. This was expressed as follows:

The system is good if it is useful.

Man, 73 years

Interpreting the test results was also perceived as difficult, including both the numbers and the charts, which made it difficult to notice if there had been any changes. A desire to receive information on how normal, healthy individuals perform in these tests compared to individuals with COPD was implied:

I know I have poor expiration values, but just how poor and compared to someone who’s healthy, you can’t see that on these graphs.

Woman, 48 years

In contrast, the system was considered *easy to use*, easy to learn, and time efficient, implying a general contentment among the participants. The information and practical guidance provided when the system was received were perceived as sufficient, since the equipment itself was regarded as rather self-explanatory. Furthermore, the questions about symptoms were considered comprehensible and quick to answer. Technical management did not cause any problems, and the few technical failures that occurred were interpreted as being caused by “misuse.” The opinion was that the system worked in the way that was expected, and it was difficult to suggest any areas for improvement:

It’s to say that the system itself worked perfectly, there were no problems.

Woman, 48 years

Participants even wished to continue using it after the study was completed.

### Affecting Technical Concern or Confidence

This category covers a process starting from uncertainty and negativity concerning technology and leading to increased technical confidence and interest. Participants´ initial uncertainty shifted to increased technical confidence and the ability to manage the system. New positive experiences and increased self-knowledge were expressed at the same time as negative experiences of uncertainty about new technology.

Participants expressed *negative feelings about technology*, describing concerns about the increasing amount of technology in society and health care. Learning new technology was perceived as difficult and new technology was even described as follows:

Terrible...I don’t know anything whatsoever about computers.

Woman, 71 years

An interest in technology was perceived as essential in order to learn new technology. However, some expressed an unwillingness to familiarize themselves with technology and a general skepticism toward new technology, particularly in relation to health care. Furthermore, the ability to master new technology was considered to decrease with increasing age. The telemonitoring system also generated thoughts on confidentiality when it came to transmitting data electronically. Sending information electronically was considered risky and concerns were raised regarding possible cyberattacks, imposters, and information leaking, and consequently, paying bills online was, for some, unthinkable.

Others reported how using the telemonitoring system *increased technical confidence*. With increasing use and familiarity, the system became easier to use and worked better:

But now I’ve got a routine for it, so I think it works really well.

Woman, 58 years

Integration of the telemonitoring system into real life seemed easier with routine. Sending data regarding health parameters electronically was considered quite harmless:

There’s information that is more sensitive...But I mean this, so what? I’ve got COPD, okay, lots of people know that.

Woman, 71 years

An attitude of wanting to “keep up” with technological development seemed to result in an increased interest in technology and, subsequently, a lower threshold for using it:

You have to learn because, even if you're not interested, you just have to learn, otherwise you won't keep up.

Woman, 48 years

Participants reported that they had considered or had already decided to purchase a tablet.

### Providing Easy Access to Health Care

A positive approach to technology marked this category. The home telemonitoring system was perceived as a promising help in existing health care, in terms of using resources properly and, subsequently, minimizing unnecessary health care visits. However, the importance of human contact was still emphasized.

The home telemonitoring system was primarily perceived as a *complement to health care*. In addition, participants appreciated the fact that the number of journeys, often perceived as long and stressful, could be decreased:

You can do it like this and then not have to go away [to the hospital or health care center].

Woman, 76 years

Distance technologies were not considered separate from ordinary care but integrated into it. Participants considered technology on the rise and visualized an opportunity to complement the telemonitoring system with video consultation, perhaps providing even better access to health care. Distance technologies were perceived to enable self-management when symptoms worsened and to provide easy communication with health care professionals. The telemonitoring system was believed to be able to help other patients with COPD in the future:

Because if you think about those who maybe live further inland and don’t have a hospital close by, then I think that it’s...They can feel more secure having a computer system like this at home.

Woman, 48 years

The *importance of human contact* was emphasized, and participants expressed the need for a health care professional who can easily be contacted for counseling and guidance. There was some concern that the increased usage of technology might result in decreased human contact:

That’s terrible. Because I want someone to talk to; I want an answer when I ask about something.

Woman, 71 years

In addition, access to a multi-professional team and being able to contact different health care professionals was desired:

That you’ve got this...that you’ve got a contact net with several people.

Woman, 58 years

However, a face-to-face contact was not the most important, but rather the knowledge that there was a human contact on the other side:

Knowing that you can get in touch either by telephone or the internet.

Man, 72 years

### Increasing Control Over the Disease

This category captures the belief that telemonitoring could provide increased security and control over the disease. When the participants felt confident in managing the system and interpreting the results, their management of the disease also improved. They believed that receiving support from health care professionals would further contribute to increased security and control.

Participants *felt reassured* by the feeling of being “looked after” by health care professionals. They did not perceive it as certain that they themselves would notice a deterioration in symptoms, but it was expected that the telemonitoring system would, which made them feel secure. Feedback from health care professionals was believed to have the potential to increase their sense of security and decrease worries about data not being transmitted. To be under surveillance was said to contribute to a feeling of being reassured, safe, and comforted. They felt reassured by someone else monitoring the physical parameters and getting in touch if there were any signs of worsening:

Yes, it would almost be nice if there’s someone there...it would give a feeling of security.

Woman, 76 years

Feedback was considered extra important at the beginning of the telemonitoring period:

I would maybe like that they sent me a graph of the results so I could see how the last month has been. Sometimes it would be nice to hear that...My god this looks really good. It works.

Woman, 58 years

The way feedback was delivered was not considered important as long as it was provided.

Participants reported an *increased awareness* of the variability of symptoms and expressed that telemonitoring could confirm how they felt on better or worse days:

I can see in clear text how things are.

Woman, 61 years

Additionally, increased confidence could result when the system confirmed the feeling of being stable in the disease:

But also when it confirms that I’m better and so on, I become more active. That I dare do more.

Woman, 58 years

However, it was experienced that the system could also cause insecurity, for example, when the saturation device showed abnormally low values. The monitoring of symptoms was perceived as making it easier for the individual to understand how the body responds to physical tasks. Most participants found monitoring their symptoms interesting and fascinating. Measuring oxygen saturation was even performed more often than necessary out of curiosity, as this parameter was already familiar and considered interesting. The system was also identified as enabling the early treatment of AEs, which was found valuable:

So you get a little hint that something’s wrong.

Man, 72 years

Consequently, increased awareness could impact on the *management of the disease*, for example, by adjusting pharmacological treatment:

I had a very high pulse during an earlier period and a low saturation level. Then I started taking cortisone and antibiotics and got better.

Woman, 58 years

The decision whether or not to seek medical help could be facilitated by the home telemonitoring system. On the other hand, some reported no change regarding the interpretation of symptoms and activities of daily living. A recurrent trait was the reluctance to seek medical help, expressed as the following:

I’ve waited almost until I’m really bad.

Woman, 58 years

The reluctance was explained by emotions, such as not wanting to be an inconvenience or to trouble health care professionals. Participants reported that their decision to seek medical care earlier could be strengthened when the system confirmed a deterioration in symptoms.

## Discussion

### Principal Findings

This study explored perceptions of the use of home telemonitoring in patients with COPD. In order to develop and implement technology that patients accept, it is important to understand barriers and enablers from the patient perspective. The main result of this study is represented by the theme *A transition toward increased control and security*. The theme symbolizes a process from insecurity and technical concerns to improved technical confidence and disease management, which is especially interesting considering that the participants did not get feedback from either the system or health care professionals. Thus, the study predominantly describes positive attitudes toward telemonitoring in patients with COPD and brings valuable information to the process of designing new or improved telemonitoring devices suitable for this important patient group.

### Interpretation of Findings

Our findings showed, despite various previous computer experiences, a general increase in technical confidence and a newfound interest in technology with participants expressing plans for purchasing new technology such as tablets. Studies on eHealth interventions in patients with COPD and other chronic conditions have found several barriers to acceptance, such as technology anxiety, a need for technical support, insufficient information, associating the use of eHealth with dependency and ill health, and concerns that the use would influence their existing health care services [27,28]. Similar to our findings, Williams et al [16] have found that, despite initial concerns, patients were able to use a telemonitoring system effectively, regardless of previous experience. As in our study, those participants also received minimal training in using the equipment prior to onset. However, there are a few differences between our study and theirs [16]. Their interviews were conducted after 6 months, instead of 3 months as in our study, which gave the participants a longer period of time to use the telemonitoring system. Their app included a symptom diary and pulse oximeter as well as multimedia educational and self-management materials [16]. Our use of a spirometer provided additional health parameters but could require extra effort on behalf of the participants. However, the findings from our study are promising since the app was mainly considered easy to use despite the extra physical effort required by the spirometer.

Increased control of the disease was part of the transition found in our study. Several other studies have also reported that telemonitoring and other eHealth solutions can improve health knowledge and produce a sense of security for patients who know that their disease is monitored [14,15,18,27,29]. In addition, the value of being under the supervision of a health care professional has been emphasized in some of these studies [15,29]. Huniche et al [15] have also found that the monitoring of symptoms confirmed the participants’ own subjective feelings, which seemed to increase their internal resources to respond to symptoms and then facilitated the decision about whether or not to seek health care. In this study, this increased awareness and security affected the participants’ self-management, which has also been found by studies of other eHealth solutions [27,29]. Since telemonitoring has been shown to have the ability to detect exacerbations [12], improved self-knowledge and self-management are important factors for decreasing health care utilization. As in this study, Korpershoek et al [30] have stated that eHealth interventions should not replace the patients’ own feelings or undermine their decisions. On the contrary, these interventions should only confirm an underlying feeling of being okay or not, thus functioning as a support to improve self-management [30]. Our participants appreciated the possibility of detecting AEs early and acting on them. This confirms findings from a study in which participants were reassured by the idea that the system could detect impending AEs and provide objective evidence to seek health care in time [17]. However, participants in this study questioned their own feelings of health when contrasting values were shown by the telemonitoring system. This is in contrast to the findings by Huniche et al [15] where the participants, instead, questioned the accuracy of the system. It is important that the patients can trust the system and receive information to guide their actions.

In addition to the transition toward increased control and security, an interesting finding in this study is the participants’ beliefs that telemonitoring and other eHealth solutions can be a good complement to existing health care. Other studies have also reported that various patient groups perceived that eHealth solutions can increase access to health care, and that these solutions can be an improvement, an alternative, or a complement to the existing health care system [27,31]. However, the importance of human contact was emphasized by our participants. This implies that when new technology is to be implemented in health care, acceptance may be greater when the technology is presented as a complement and not as a replacement of personal care, which is in agreement with previous studies of various patient groups [30-32]. An important enabler for the effectiveness of telemonitoring in managing COPD is a patient-provider relationship [33]. Continuity and direct contact with a health care professional who knew the patient and gave a prompt response to symptom deterioration was crucial for our participants’ sense of security. This has also been reported earlier in studies evaluating telemonitoring interventions that included consultations with the study center [19,29]. In order to facilitate this relationship, our participants requested video consultations as a complement to the telemonitoring system. Nissen and Lindhardt [29] have reported that patients with COPD were more relaxed and focused and felt more secure during video consultations than with visits at the outpatient clinic. In contrast, a study from 2006 [34] has reported that participants expressed a sense of alienation during video consultations, as well as problems with the patient-doctor communication, in that they felt they had not been seen and that the consultation felt artificial. However, that study was published in 2006, and technological equipment is now much more common. The use of the internet, computers, and smartphones has dramatically increased [35,36], which may suggest that the general population of today is more accustomed to video calls than in 2006. A recent review by Kruse et al [33] on telemonitoring to manage COPD states that as more service options have been added to telemonitoring devices, including video consultation and phone support, a reduction in hospital admissions due to AEs has been reported.

Our participants suggested that the telemonitoring system could result in better access to health care and replace visits to a health care setting. This would minimize strenuous travel and would benefit patients living in rural areas. This is in agreement with the results of other eHealth studies suggesting that enhanced access to care is especially useful in rural areas with restricted access to health care settings [37,38]. The need to travel has been identified as a barrier for patients with COPD, due to poor mobility, lack of transport, and cost of travel [39-41]. There may also be an environmental aspect involved. Telemonitoring generates far fewer carbon emissions than traditional health care, where patients have to travel to hospitals or health care centers [42]. Therefore, making telemonitoring a natural complement to traditional health care can make an important contribution to reducing the greenhouse gas emissions fueling climate change.

In summary, in addition to the transition from insecurity to confidence and control, the participants in this study expressed both negative and positive attitudes and emotions regarding telemonitoring. A review by Kruse et al [33] has identified multiple factors as both enablers and barriers, which further illustrates this complexity. This complexity indicates that telemonitoring, and perhaps eHealth solutions in general, should be tailored to fit different patients and marketed with different strategies in order to achieve successful implementation. Another study performed in Sweden indicates that an important factor for successful implementation is that the system should meet the patients’ perceived needs and fit their self-image [43]. Therefore, including potential users at an early stage in the development process, so-called co-design [44], could be beneficial [45]. A recent study by Tistad et al [46] concludes that the involvement of user groups can strengthen the potential for a system to be adopted into everyday life and clinical practice. With this in mind, a weakness with our telemonitoring system is that the participants were not involved in its development. Perhaps negative emotions and feelings of concern and insecurity could have been reduced by using co-design.

### Strengths and Limitations

We strove for trustworthiness [20] in several ways during the process of data collection and analysis and with the description of this study. First, we tried to obtain a maximum variation sample in the process of ensuring credibility [20]. However, the recruiting process was difficult, especially when it came to achieving gender balance, and fewer men were included. It was difficult to recruit men to the telemonitoring project; they had a greater tendency to decline participation than women. Therefore, toward the end, more efforts were made to include more men in order to equalize the gender balance. The reason why more men declined is unknown. This contradicts two previous studies in which there was either no significant gender difference [47] or more females than males who declined participation [48]. A majority of the interviews were 25-45 minutes long, and all of them contributed to the depth of the material. Some of the interviews were conducted by telephone for practical reasons. Several previous studies have concluded that telephone interviews are valuable for data collection and can be equated with face-to-face interviews [49-51]. Discussions about the analysis were performed repeatedly among researchers with varying competence, methodological backgrounds, and insider and outsider perspectives, which is a strength of this study. A semistructured interview guide was used in order to increase dependability [20]. To obtain transferability [20], we have presented a thorough description of the method used and justifications of its use, and we have fulfilled the standards required for reporting qualitative research [21]. Results are presented in text, in tables, and with supportive quotes, which also enhance transferability [20].

### Implications

This study’s results imply that there are many perceived enablers as well as barriers to the implementation of telemonitoring for patients with COPD. It is important not to withhold information; the participants must understand the aim of the system and be fully informed and educated about it. Appropriate training and access to prompt support is necessary to alleviate technical insecurity.

The results of this study can hopefully assist future research on telemonitoring, particularly in the design of user-friendly systems, as well as tools to predict which patients are most likely to find the equipment useful. This study has contributed to illuminating the user perspective on a telemonitoring system, which is an important perspective in the process of successful implementation. The results of this study also indicate that telemonitoring may have a potential role as a valuable complement to existing health care. It is known that patients with COPD do not receive the recommended health care [39,52]. Considering this, it would be unethical not to further explore eHealth solutions, such as telemonitoring, since it can contribute to more equity in health care. However, it is also important to consider that eHealth is not suitable for all patients. Other solutions to complement existing health care are also needed to reduce inequity in health care.

In summary, additional research is needed regarding telemonitoring and other eHealth solutions and their implementation, bearing in mind the complexity of the enablers and barriers. The use of co-design is a promising method. It is also necessary to examine whether the use of eHealth solutions is influenced by individual factors, such as gender, ethnicity, age, and technical experience among people with COPD. These factors have been shown to influence technology use in general [53].

### Conclusions

Participants in this study described various perceptions of using telemonitoring. The results implicate a transition toward increased control and security with telemonitoring, leading to increased self-knowledge, a sense of security, and improved self-management. However, concerns were raised about technical errors, insufficient information, and the ability to cope with technology. In order to further improve the development and implementation of telemonitoring systems, several actions are needed, such as improved patient education and the use of co-design where the users are involved in the development. Furthermore, telemonitoring should be viewed as a complement to existing health care, bearing in mind the importance of human contact. If correctly implemented, telemonitoring has the potential to contribute to earlier health care contacts and, thereby, the early detection of COPD AEs, improved self-management, and equity in care for patients with COPD.

## Abbreviations

AE: acute exacerbation
CAT: chronic obstructive pulmonary disease assessment test
COPD: chronic obstructive pulmonary disease
eHealth: electronic health
FEV1: forced expiratory volume in 1 second
GOLD: Global Initiative for Chronic Obstructive Lung Disease
HADS: Hospital Anxiety and Depression Scale
IC: inspiratory capacity
mMRC: modified Medical Research Council
